# GRP75 mediates endoplasmic reticulum–mitochondria coupling during palmitate-induced pancreatic β-cell apoptosis

**DOI:** 10.1016/j.jbc.2021.101368

**Published:** 2021-10-29

**Authors:** Shweta Tiwary, Arun Nandwani, Rukshar Khan, Malabika Datta

**Affiliations:** 1 Integrative and Functional Biology Group, CSIR-Institute of Genomics and Integrative Biology, Mall Road, Delhi, India; 2 Academy of Scientific and Innovative Research (AcSIR), Ghaziabad, India; 3 Department of Biosciences, Jamia Millia Islamia, New Delhi, India

**Keywords:** palmitate, GRP75, ER–mitochondria contact, calcium apoptosis, BiP, binding immunoglobulin protein, BSA, bovine serum albumin, CHOP, C/EBP homologous protein, ER, endoplasmic reticulum, FACL4, fatty acid-CoA ligase type4, GRP75, glucose regulated protein 75, IP3R, inositol 1,4,5 trisphosphate receptor, ITT, insulin tolerance test, MAM, mitochondria-associated membrane, MFN1, mitofusin1, MFN2, mitofusin2, OGTT, oral glucose tolerance test, PACS2, phosphofurin acidic cluster sorting protein 2, PLA, proximity ligation assay, TEM, transmission electronic microscopy, VDAC, voltage-dependent anion channel

## Abstract

The endoplasmic reticulum (ER) and mitochondria are structurally connected with each other at specific sites termed mitochondria-associated membranes (MAMs). These physical links are composed of several tethering proteins and are important during varied cellular processes, such as calcium homeostasis, lipid metabolism and transport, membrane biogenesis, and organelle remodeling. However, the attributes of specific tethering proteins in these cellular functions remain debatable. Here, we present data to show that one such tether protein, glucose regulated protein 75 (GRP75), is essential in increasing ER–mitochondria contact during palmitate-induced apoptosis in pancreatic insulinoma cells. We demonstrate that palmitate increased GRP75 levels in mouse and rat pancreatic insulinoma cells as well as in mouse primary islet cells. This was associated with increased mitochondrial Ca^2+^ transfer, impaired mitochondrial membrane potential, increased ROS production, and enhanced physical coupling between the ER and mitochondria. Interestingly, GRP75 inhibition prevented these palmitate-induced cellular aberrations. Additionally, GRP75 overexpression alone was sufficient to impair mitochondrial membrane potential, increase mitochondrial Ca^2+^ levels and ROS generation, augment ER–mitochondria contact, and induce apoptosis in these cells. *In vivo* injection of palmitate induced hyperglycemia and hypertriglyceridemia, as well as impaired glucose and insulin tolerance in mice. These animals also exhibited elevated GRP75 levels accompanied by enhanced apoptosis within the pancreatic islets. Our findings suggest that GRP75 is critical in mediating palmitate-induced ER–mitochondrial interaction leading to apoptosis in pancreatic islet cells.

Eukaryotic cells possess a complex network among various intracellular compartments and organelles, in addition to each executing their specific own functions. Although they act independent of each other, yet an interaction or cross talk among them is integral in cell function and survival (1). This often occurs at the contact sites between their individual membranes, where two involved organelles are closely positioned without undergoing fusion (2, 3). Among these, the most prominent and well-studied membrane contact sites are those between the endoplasmic reticulum (ER) and the mitochondria. The close contact sites between the ER and the mitochondria are termed as MAMs (mitochondria-associated membrane proteins) (4) with their main function being control of lipid biosynthesis, mitochondrial division, Ca^2+^ signaling, maintenance of coordinated dynamics between the organelles and apoptosis (5). The physical tethering of ER and mitochondria is achieved by interaction between proteins and protein complexes. Some of these include linkages between the mitochondrial voltage-dependent anion channel (VDAC) and the ER membrane resident protein, IP_3_R through a cytosolic protein, glucose regulated protein 75 (GRP75) (6), homotypic and heterotypic interactions between the ER-bound mitofusin2 [MFN2] and the mitochondrial mitofusin1 [MFN1] or MFN2 (7, 8). Sigma-1R, an ER resident protein, also localizes at MAM and forms a calcium-sensitive chaperone complex with binding immunoglobulin protein (BiP) (Grp78) (9). Multiple phospholipid and glycosphingolipid-synthesizing enzymes, which include long-chain-fatty acid-CoA ligase type4 (FACL4) and phosphatidylserine synthase-1 (PSS-1), are also present at these sites (10).

Considering the significance of the ER–mitochondria interaction in mediating diverse cellular functions, it is but expected that impaired ER–mitochondria tethering might destabilize cellular functions and give rise to an array of diseases. Aberrant ER–mitochondria cross talk and deregulated levels of MAM proteins have been associated with various diseases including obesity, diabetes, cancer, and neurodegenerative disorders such as Alzheimer’s disease, Parkinson’s disease, and Amyotrophic Lateral Sclerosis (11, 12, 13). ER–mitochondria interaction is altered in livers of both, ob/ob and diet-induced insulin-resistant mice (14), which results in mitochondrial calcium overload, disrupted oxidative capacity of the mitochondria, and higher oxidative stress. Forced downregulation of ER–mitochondria tethering proteins improves mitochondrial oxidative capacity and glucose metabolism in obese animals (15). PDK4 (pyruvate dehydrogenase kinase 4) interacts with the IP_3_R-GRP75-VDAC1 complex at the MAM surface, thereby influencing insulin signaling in skeletal muscles along with mitochondrial Ca^2+^ accumulation, dysfunctional mitochondria, and induction of ER stress (16). Disruption of ER–mitochondria interaction is an early event prior to mitochondrial dysfunction and insulin resistance (17), and this significantly lowers organelle interaction as is seen in human pancreatic sections from diabetic donors (18). Chronic incubation of pancreatic islets with high glucose alters GSIS (glucose-stimulated insulin secretion) accompanied with reduced ER calcium, increased basal mitochondrial calcium, reduced ATP-stimulated ER–mitochondria calcium exchanges, and increased organelle interaction, all contributing to glucotoxicity-induced β-cell death (19).

Several causes are identified that lead to impaired ER–mitochondria cross talk and consequently deregulate normal cellular physiological dynamics. Obesity that is accompanied with increased circulatory fatty acids is a major phenotype frequently associated with such impairment (17, 20, 21, 22). Among the circulatory free fatty acids that are increased during obesity, palmitate is the most abundant saturated fatty acid (23) and is identified as a causative factor of several cellular aberrations associated with obesity (24). Palmitate-induced pancreatic β-cell apoptosis is one of the critical factors attributable to the progression of obesity and insulin resistance to type 2 diabetes (25, 26). Long-term exposure to palmitate impairs GSIS (glucose-stimulated insulin secretion) and promotes pancreatic β-cell death (27).

Interestingly, in the liver, palmitate has been shown to reduce MAM contact area and decrease the functional interaction between the ER and the mitochondria (28). Forced overexpression of MFN2, a critical component of the MAM, partially restored MAM contact area and ER–mitochondria interactions (28). While these suggest a role of palmitate in destabilizing ER–mitochondria interaction, not much has been reported of its role in influencing this interaction in the pancreas where ER–mitochondria cross talk is a significant event that is linked to pancreatic apoptosis as seen in several diseases including diabetes.

Here, we sought to study the contribution of palmitate in impairing ER–mitochondria contact, the roles of MAM proteins, and the consequent effects on pancreatic islet function.

## Results

### Palmitate increases GRP75 levels in MIN6 cells

**Figure 1:**
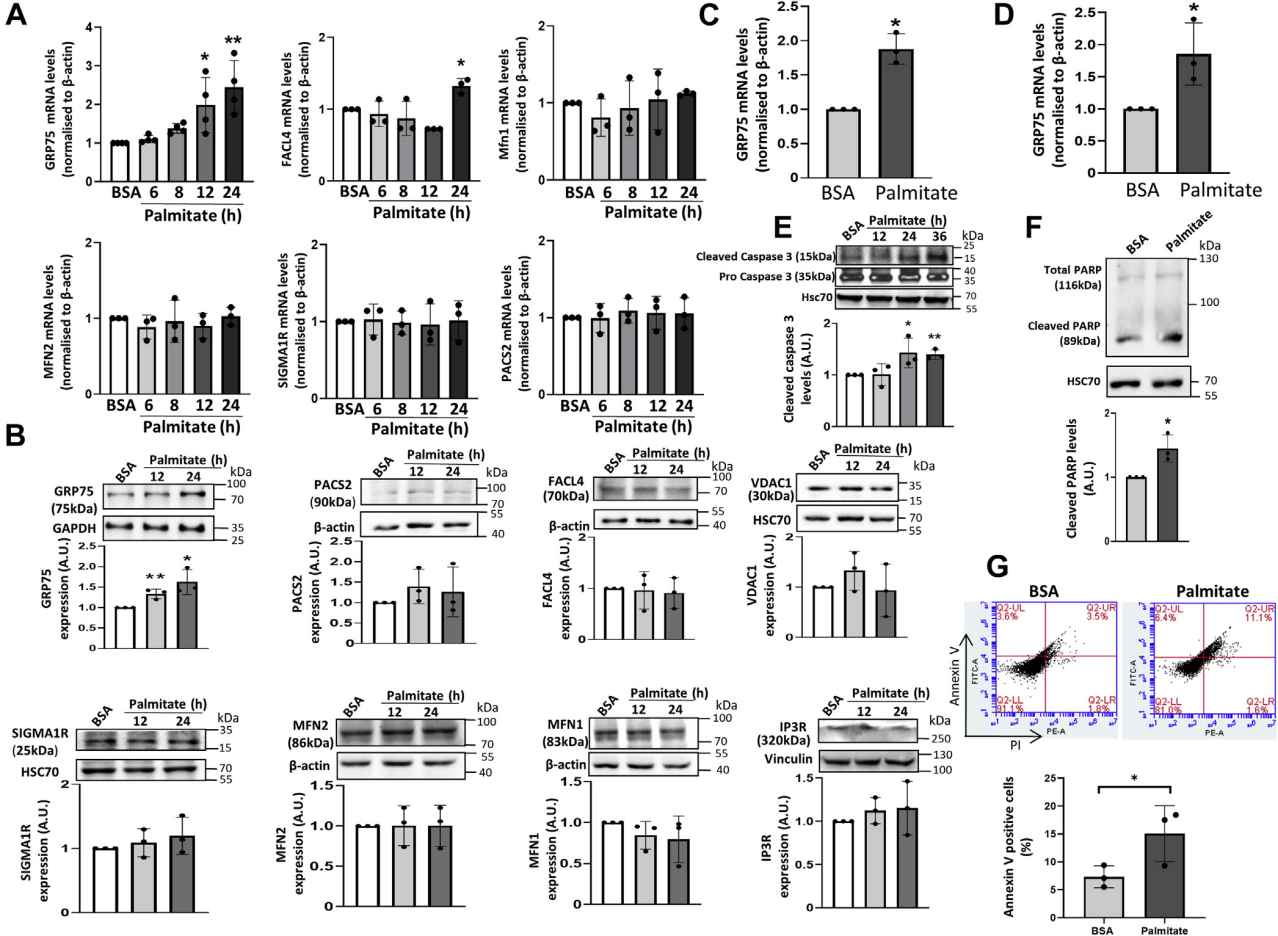
**Palmitate treatment increases GRP75 levels.***A*, MIN6 cells were treated with palmitate (0.4 mM) or BSA for different time points. On termination of incubation, RNA was isolated and assessed for the transcript levels of GRP75, FACL4, MFN1, MFN2, PACS2, and Sigma1R by qRT-PCR using specific primers. β-actin was used as the normalization control. *B*, MIN6 cells treated with BSA or palmitate were lysed, and 20 to 50 μg protein was subjected to Western blot analysis to assess levels of MAM proteins. GAPDH or vinculin or HSC70 or β-actin was used as the loading control. A representative blot is given and the densitometric analysis in palmitate and BSA-treated cells is shown. RINm5F (*C*) and mouse primary islets (*D*) were incubated in the presence of either BSA or palmitate (0.4 mM) for 24 h and the transcript levels of GRP75 were assessed by qRT-PCR. *E*, MIN6 cells were treated with palmitate or BSA for different time points and lysates (50 μg) were subjected to Western blot analysis using anti-caspase 3 antibody. HSC70 was used as a loading control. *F*, MIN6 cells were incubated in the presence of either BSA or palmitate (0.4 mM) and on termination of incubation (36 h), cells were evaluated for induction of apoptosis by detecting cleaved PARP levels by Western Blot analysis using 50 μg protein (HSC70 was taken as the loading control) and by labeling with Annexin V and propidium iodide and analyzing by flow cytometry with Annexin V positive cells being presented (*G*). All experiments were performed at least thrice, and values are reported as the means ± SD. ∗*p* < 0.05, ∗∗*p* < 0.01 as compared with BSA-treated cells. GRP75, glucose regulated protein 75; MFN1, mitofusin1; MFN2, mitofusin1; PACS2, phosphofurin acidic cluster sorting protein 2.

**Figure 2:**
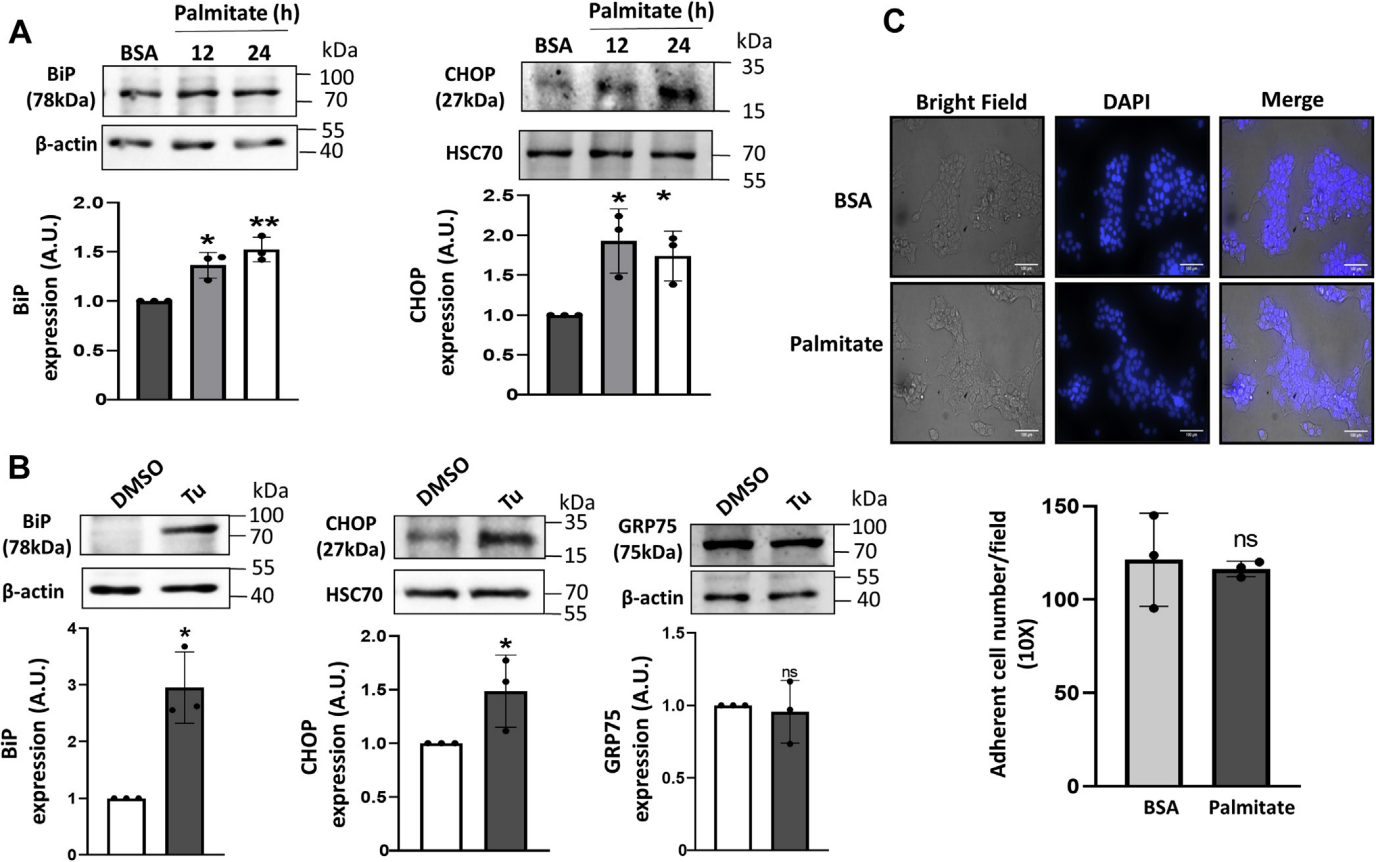
**Palmitate induces ER stress but does not alter cell numbers in MIN6 cells.***A*, MIN6 cells were seeded in six-well plates and treated with either BSA or palmitate (0.4 mM) and on termination of incubation (12 and 24 h), cells were lysed, and 30 μg protein was analyzed for the levels of binding immunoglobulin protein (BiP) and C/EBP homologous protein (CHOP) by Western Blot analysis. A representative blot is shown and densitometric analysis of three independent experiments is given below. *B*, MIN6 cells were incubated for 8 h in the presence of either DMSO or Tunicamycin (Tu, 5 μg/ml), lysed, and 30 μg protein was subjected to Western Blot analysis for the detection of BiP, CHOP, and glucose regulated protein 75 (GRP75). Representative blots are given and below are the respective densitometric analyses. *C*, MIN6 cells incubated with either BSA or palmitate (0.4 mM) for 36 h were stained with DAPI, and images were captured in a fluorescent microscope. Individual nuclei of adherent cells from randomly selected fields were counted (scale bars: 100 μm). Values are reported as the means of three independent replicates ±SD. ∗*p* < 0.05, ∗∗*p* < 0.01 as compared with BSA or DMSO -treated cells.

We had previously shown that during pancreatic β-cell apoptosis, there is ER and mitochondria dysfunction accompanied by increased transfer of calcium from the ER to mitochondria (29, 30). In this study, we examined the status and function of proteins at the interface of the ER and the mitochondria (MAM) during palmitate-induced pancreatic apoptosis. Mouse pancreatic insulinoma cells (MIN6) were treated with palmitate (0.4 mM) at different time points and the transcript levels of MAM proteins were assessed by qRT PCR. There was a time-dependent increase in the transcript levels of GRP75 starting from 12 h of palmitate treatment, along with modest increases in FACL4 mRNA levels at 24 h. However, there was no change in transcript levels of the other MAM proteins, namely MFN1, MFN2, phosphofurin acidic cluster sorting protein 2 (PACS2), and SIGMA1R (Fig. 1*A*). Such changes in mRNA levels were validated at the protein level and as in the transcript changes, GRP75 protein levels showed a significant increase in the presence of palmitate (Fig. 1*B*). Palmitate did not alter the protein levels of the other MAM proteins. This was validated in rat islet derived insulinoma cells, RINm5F (Fig. 1*C*) and mouse primary islets (Fig. 1*D*) where palmitate significantly increased GRP75 transcript levels. Concomitantly, palmitate-induced apoptosis was evident by elevated cleaved caspase 3 and cleaved PARP levels and increase in annexin V positive cell levels (Fig. 1, *E*–*G*). Palmitate-induced apoptosis is often preceded by induction of ER stress, and our data demonstrated that palmitate (0.4 mM) increased the protein levels of the ER stress markers, BiP and C/EBP homologous protein (CHOP) (Fig. 2*A*). This suggested that palmitate-induced increase in GRP75 as in Figure 1, *A* and *B* might be mediated by the ER stress response. Nevertheless, while incubation of MIN6 cells with an ER stress inducer, tunicamycin (5 μg/ml; 8 h) induced ER stress within the cells as evidenced by increased protein levels of BiP and CHOP, there was no significant change in the protein levels of GRP75 (Fig. 2*B*), suggesting that induction of GRP75 levels can be achieved without the mediation of ER stress. Therefore, although palmitate induces both, ER stress and GRP75 levels in MIN6 cells, the induction of GRP75 is possibly not dependent on palmitate-mediated ER stress induction. However, despite these events, as compared with bovine serum albumin (BSA)-treated cells, the cell number was unchanged by palmitate suggesting that palmitate did not exert any change in the adherent cell number at the dose used (Fig. 2*C*), and therefore, while palmitate did initiate and induce apoptosis in MIN6 cells, this did not translate into the cell number yet being affected at 36 h of palmitate incubation.

### GRP75 inhibition abrogates palmitate-induced apoptosis

**Figure 3:**
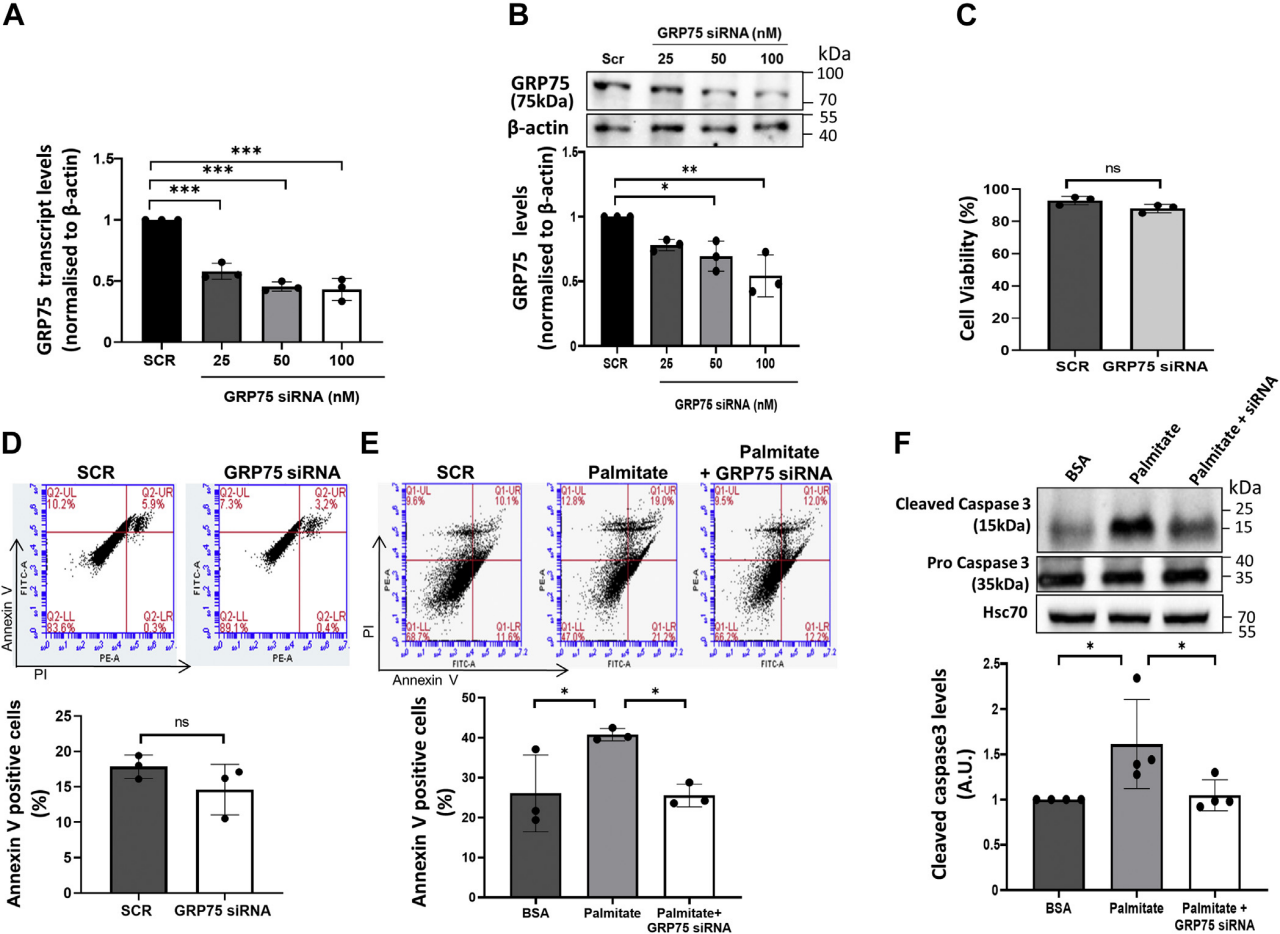
**GRP75 inhibition abrogates palmitate-induced apoptosis.***A*, MIN6 cells were transfected with the scramble (SCR) or with GRP75 siRNA (25–100 nM), and after 24 h, RNA was isolated and assessed for the transcript levels of GRP75 by qRT-PCR using specific primers. β-actin was used as normalization control. *B*, MIN6 cells were transfected as described above, lysed after 48 h, and the levels of GRP75 were evaluated by Western blot. β-actin was used as normalization control. *C*, cell viability in the presence of the scramble (SCR) or GRP75 siRNA was analyzed using MTT (3-(4,5-Dimethylthiazol-2-yl)-2, 5-diphenyltetrazolium bromide, a tetrazole), and absorbance was measured on microtiter plate reader and normalized to the total number of cells. *D*, MIN6 cells transfected with either the scramble or GRP75 siRNA (100 nM) for 48 h were washed and labeled with annexin V and Propidium Iodide (PE-A) and analyzed for apoptosis by flow cytometry. Percent of annexin V positive cells is presented. *E*, MIN6 cells were transfected with either scramble or GRP75 siRNA (100 nM) and after 24 h, they were incubated in the absence or presence of palmitate (0.4 mM for 36 h) and on completion of incubation, cells were washed and labeled with annexin V and Propidium Iodide (PE-A) and analyzed for apoptosis by flow cytometry. Annexin V (FITC-A) positive cells for each incubation is presented. *F*, MIN6 cells were incubated with palmitate with or without GRP75 siRNA and lysed. Levels of cleaved caspase 3 and caspase 3 were analyzed by Western blot using anti-caspase 3 antibody. HSC70 was used as loading control. All experiments were performed at least thrice, and values are reported as the mean ± SD. ∗*p* < 0.05, ∗∗*p* < 0.01, ∗∗∗*p* < 0.001. GRP75, glucose regulated protein 75.

GRP75 is critical in maintaining a physical connect between the ER and the mitochondria along with IP_3_R and VDAC (6). The results above suggest that the observed deregulation of GRP75 might be critical in palmitate-induced apoptosis by modulating ER–mitochondria interactions. We sought to evaluate if GRP75 inhibition could rescue palmitate induced apoptosis in MIN6 cells. GRP75 levels in MIN6 cells were inhibited using a specific siRNA (25-100 nM) wherein GRP75 levels were significantly inhibited, both at the transcript and protein levels (Fig. 3, *A* and *B*). GRP75 siRNA, at the dose and for the time period used, did not exert any significant detrimental effects on cell viability or apoptosis (Fig. 3, *C* and *D*). However, while palmitate exhibited a significant increase in annexin V positive (apoptotic) cells as compared with BSA-treated cells, these were significantly prevented by GRP75 inhibition (Fig. 3*E*). Also, cleaved caspase 3 protein levels were significantly increased, by almost 1.5-fold in the presence of palmitate, and this was significantly prevented by GRP75 siRNA (Fig. 3*F*). All these suggest that GRP75 is a critical mediator of palmitate-induced apoptosis in these cells.

### GRP75 inhibition attenuates palmitate-induced deregulated calcium movements between the ER and mitochondria

**Figure 4:**
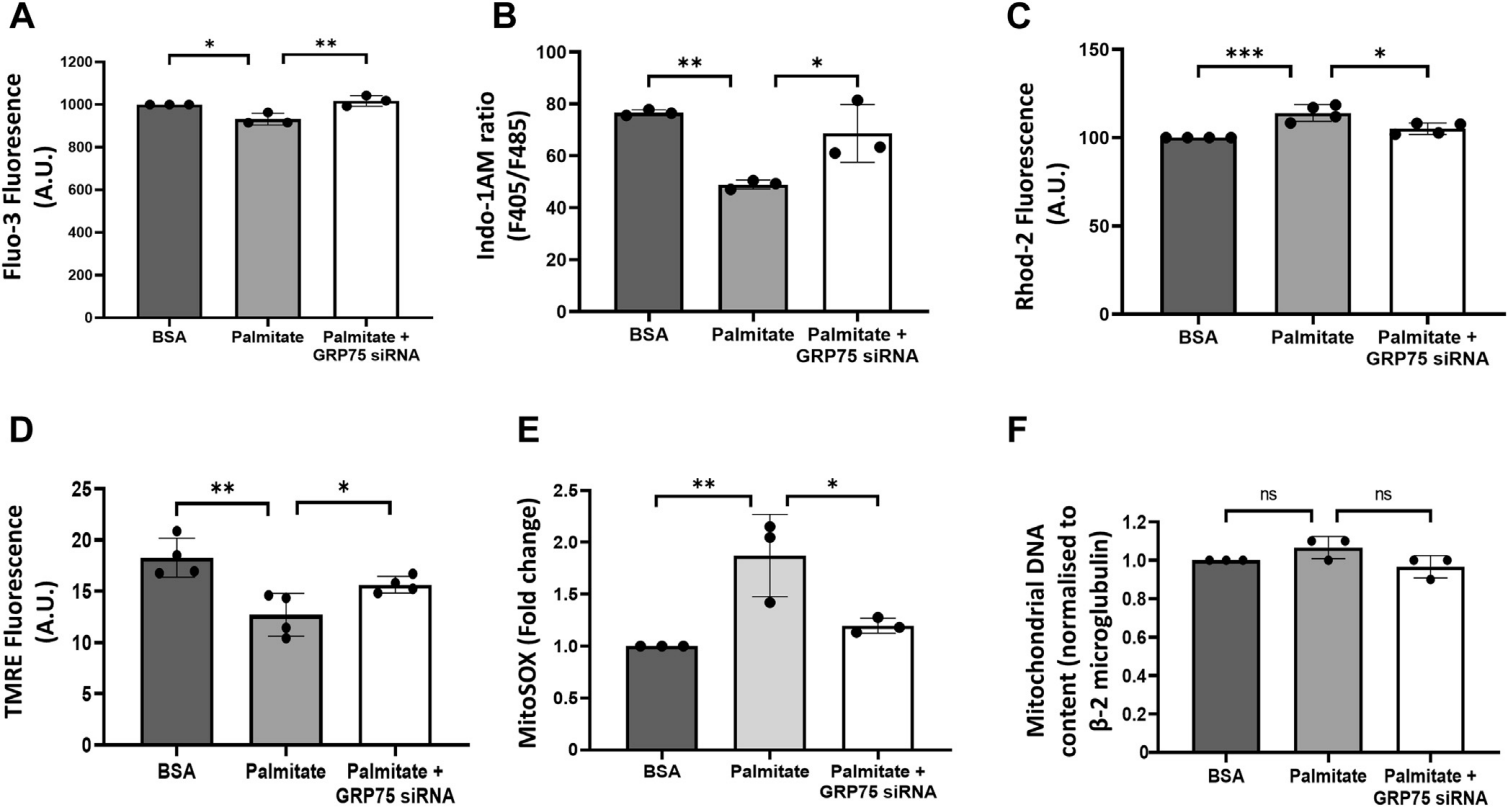
**GRP75 inhibition attenuates palmitate-induced deregulated calcium movements between the ER and mitochondria.** MIN6 cells transfected with either the scramble or GRP75 siRNA (100 nM) were then incubated with palmitate (0.4 mM) for 24 h. On termination of incubation, cells were assessed for the levels of intracellular (*A* and *B*) and mitochondrial (*C*) calcium levels using Fluo-3, Indo-1AM, and Rhod-2. Fluorescence values for Fluo-3 and Rhod-2 and ratio of fluorescence at 405 nm (F405) and 485 nm (F485) for Indo-1AM are presented. *D*, MIN6 cells were treated as described above and on termination of incubation, mitochondrial membrane potential was measured by flow cytometry using TMRE (Tetramethylrhodamine, Ethyl Ester) dye. *E*, MIN6 cells transfected with scramble with GRP75 siRNA were then treated with palmitate (0.4 mM). On completion of incubation, cells were incubated for 10 min at 37 °C with 5 μM Mitosox fluorescent marker and analyzed by flow cytometry for endogenous reactive oxygen species content. *F*, MIN6 cells incubated as in (*A*) were assessed for mitochondrial number using d-loop specific primers by qRT-PCR. β-2 microglobulin was used as normalization control. All experiments were performed at least thrice and values are reported as the means ± SD. ∗*p* < 0.05, ∗∗*p* < 0.01, ∗∗∗*p* < 0.001. GRP75, glucose regulated protein 75.

The onset of the apoptotic cascade within cells is preceded by calcium movements into the mitochondria, and since palmitate-induced apoptosis was abrogated by GRP75 inhibition, we sought to determine the role of GRP75 inhibition on cellular calcium movements in the presence of palmitate. Palmitate treatment decreased cytosolic calcium levels, and this decrease was prevented by GRP75 inhibition (Fig. 4, *A* and *B*). Concomitantly, mitochondrial calcium levels, as measured by Rhod-2, significantly increased after palmitate induction, which was attenuated in the presence of GRP75 siRNA (Fig. 4*C*). Such increased load of calcium within the mitochondria interferes with its function and promotes mitochondrial dysfunction. Palmitate significantly impaired mitochondrial membrane potential and also increased endogenous ROS production (Fig. 4, *D* and *E*); these were significantly prevented in the presence of GRP75 siRNA, suggesting an important role of GRP75 in palmitate-induced mitochondrial dysfunction. To substantiate that such mitochondrial deregulation was in fact due to impaired mitochondrial function and not due to changes in mitochondrial numbers, we determined the mitochondrial number with the help of mitochondrial D-loop primers. There was no significant change in mitochondrial numbers either with palmitate or with GRP75 siRNA as compared with control (Fig. 4*F*).

### Overexpression of GRP75 sensitizes MIN6 cells to apoptosis and mitochondrial dysfunction

**Figure 5:**
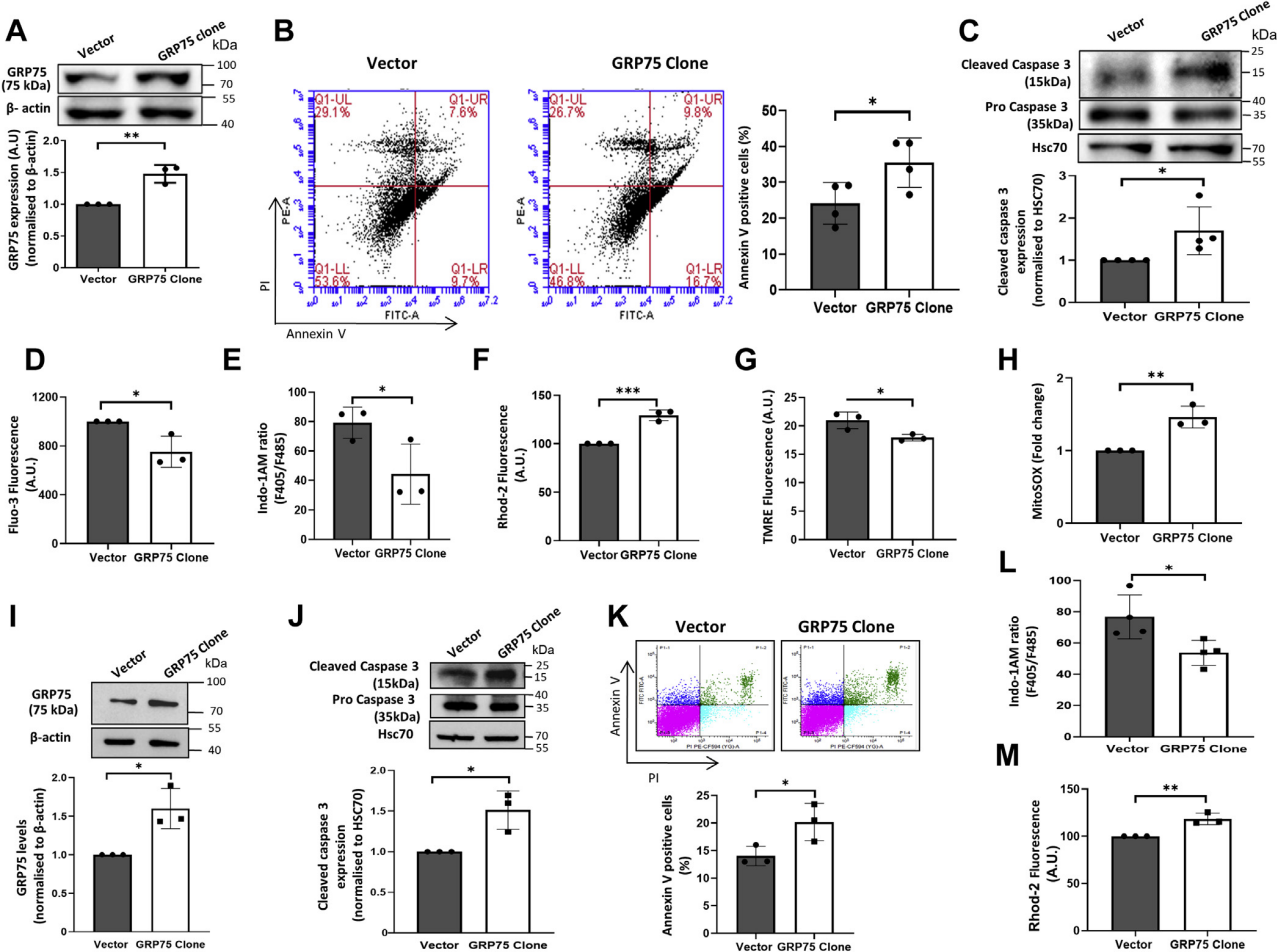
**Overexpression of GRP75 promotes apoptosis and mitochondrial dysfunction in MIN6 cells.***A*, MIN6 cells transfected with the empty vector or with the GRP75 overexpression vector (2 μg) were lysed after 48 h of incubation and 30 μg protein was used for Western blot analysis using anti-GRP75 antibody. β-actin was used as a loading control. *B*, MIN6 cells transfected as in (*A*) were incubated with annexin V (FITC-A) and propidium iodide (PE-A) for 15 min at room temperature and analyzed for apoptosis by flow cytometry. *C*, MIN6 cells transfected with either the empty vector or the GRP75 clone (48 h) were lysed, and 50 μg protein was analyzed for the levels of cleaved caspase 3, by Western Blot analyses. MIN6 cells transfected as in (*A*) were loaded with Fluo-3 (*D*), Indo-1AM (*E*), and Rhod-2 (*F*) and analyzed for cytosolic and mitochondrial calcium levels. MIN6 cells transfected with the empty vector or the GRP75 overexpression vector were evaluated for mitochondrial membrane potential (*G*) and ROS generation (*H*). *I*, MIN6 cells were transfected with either the empty vector or the tetracycline inducible pcDNA4/TO/HisA-Grp75 clone (2 μg) and after induction with tetracycline for 48 h, cells were lysed and the levels of GRP75 (*I*) and cleaved caspase 3 (*J*) were evaluated by Western Blot analysis. β-actin and HSC70 were taken as the loading controls. MIN6 cells transfected and induced with tetracycline as in “*I*” were incubated with annexin V (FITC-A) and propidium iodide (PE-A) and analyzed for apoptosis by flow cytometry (*K*) and cytosolic Ca^2+^ was measured using Indo-1AM (*L*) and mitochondrial Ca^2+^ using Rhod 2 (*M*). All experiments were done at least thrice and values are the means ± SD. ∗*p* < 0.05, ∗∗*p* < 0.01, ∗∗∗*p* < 0.001 as compared with the empty vector. GRP75, glucose regulated protein 75.

Results until now demonstrate a significant role of GRP75 in palmitate-induced mitochondrial dysfunction and apoptotic cell death. To further authenticate the primary role of GRP75 in mitochondrial dysfunction leading to cell death, MIN6 cells were transiently transfected with a GRP75 overexpression vector or an empty vector. With a transfection efficiency of approximately 70%, GRP75 overexpression caused a significant increase in GRP75 protein levels (Fig. 5*A*), and this increase alone was sufficient to significantly induce apoptosis as compared with empty-vector transfected cells (Fig. 5*B*). This was also corroborated by the fact that GRP75 overexpression was adequate enough to elevate cleaved caspase 3 levels (Fig. 5*C*), decrease cytosolic calcium levels (Fig. 5, *D* and *E*), increase mitochondrial calcium concentration (Fig. 5*F*), significantly impair mitochondrial membrane potential (Fig. 5*G*), and increase mitochondrial ROS generation (Fig. 5*H*). To further confirm these results, MIN6 cells were transfected with either the empty vector or the tetracycline inducible pcDNA4/TO/HisA vector containing the GRP75 insert and the repressor pcDNA6/TR6 vector. At 48 h of induction with tetracycline, there was a significant increase in GRP75 levels (Fig. 5*I*) as compared with the empty-vector transfected cells, and this was accompanied by elevated levels of cleaved caspase3 and increased numbers of annexin V positive cells, decreased cytosolic Ca^2+^ levels, and increased mitochondrial Ca^2+^ levels (Fig. 5, *J*–*M*). Taken together, these data suggest an important role of GRP75 in mediating deregulated calcium movements, mitochondrial dysfunction, and consequently apoptotic cell death in these cells.

### Palmitate increases ER–mitochondria proximity in MIN6 cells

**Figure 6:**
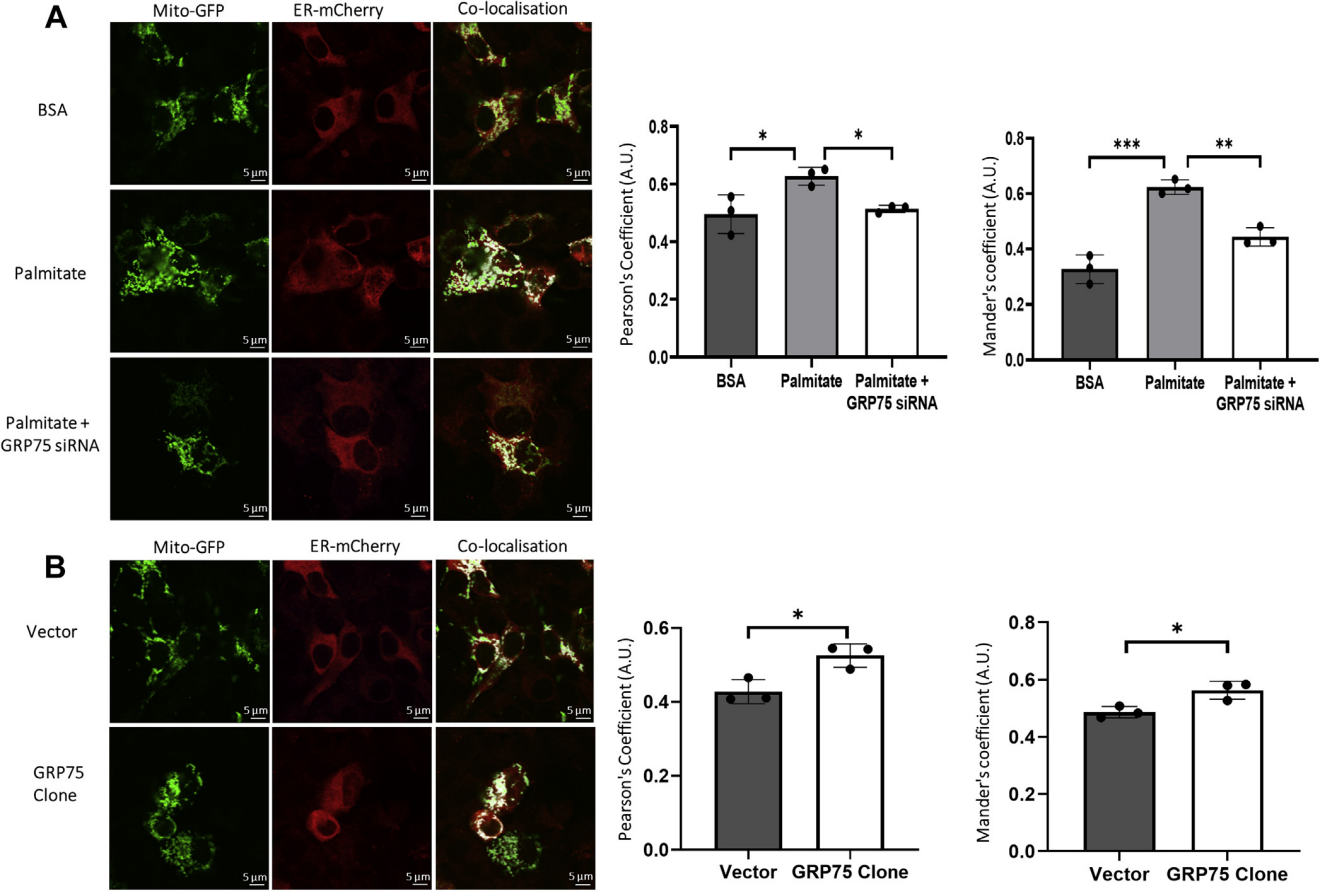
**Palmitate treatment increases ER–mitochondria proximity in MIN6 cells.***A*, MIN6 cells cotransfected with either the scramble or glucose regulated protein 75 (GRP75) siRNA and mito-GFP and ER-mcherry (1 μg) were incubated in the presence of BSA or with palmitate (0.4 mM). Expression of mito-GFP and ER-mCherry was imaged by confocal microscopy (scale bars: 5 μm). Mander’s and Pearson’s coefficients for each incubation were calculated by taking an average of data obtained for all the z-stacks (n = 45–60) of each cell. *B*, MIN6 cells were transfected with the empty vector and the GRP75 overexpression vector (2 μg, 48 h) along with mito-GFP and ER-mCherry and imaged in a confocal microscope. Mander’s and Pearson’s coefficients shown (n = 65–70) were calculated as in (*A*). Results shown are the means ± SD of three independent experiments; ∗*p* < 0.05; ∗∗*p* < 0.01; ∗∗∗*p* < 0.001.

**Figure 7:**
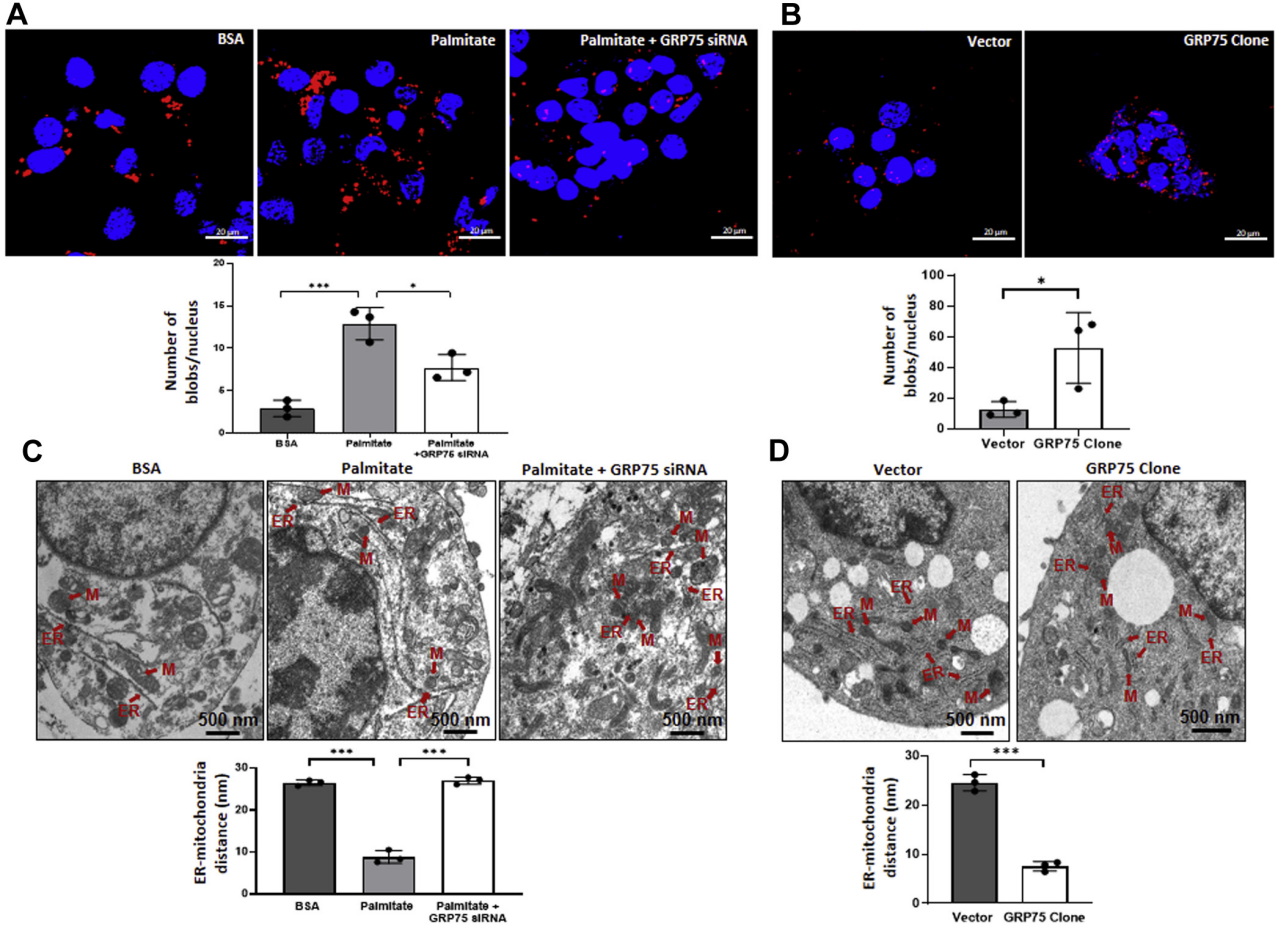
**Palmitate and GRP75 overexpression regulates ER–mitochondria interaction.***A*, MIN6 cells transfected with either the scramble or GRP75 siRNA were incubated in the presence of palmitate (0.4 mM) for 36 h. On termination of incubation, cells were subjected to *in-situ* proximity ligation assay using anti-VDAC1 and anti-IP3R antibodies. Representative confocal images of the cells are shown where interaction between IP3R and VDAC1 due to proximity ligation is indicated by *red dots*. Nuclei are indicated by blue staining using DAPI. Scale: 20 μm. Quantification of dots per nucleus is shown below. At least five images were captured per incubation. *B*, MIN6 cells were transfected with the empty vector or with the GRP75 overexpression vector and after 48 h, cells were analyzed for proximity between VDAC1 and IP3R as in “*A*” (Scale bars: 20 μm). Representative confocal images are given and the quantification of the interaction blobs (*red dots*) per nucleus is shown below. *C*, MIN6 cells transfected and incubated as in “*A*” were fixed in glutaraldehyde, embedded, sectioned, stained, and visualized in a transmission electron microscope. Representative figures (magnification 13,500×; scale: 500 nm) are given and the quantified distance between the endoplasmic reticulum (ER) and the mitochondria (M) is given below. *Arrows* show the ER and the mitochondria. *D*, transmission electron microscope images (TEM) of MIN6 cells transfected with the empty vector or with the GRP75 overexpression vector; representative TEM images (13,500×; scale bars: 500 nm) are given, and the quantified distance between the endoplasmic reticulum (ER) and the mitochondria (M) is given below. *Arrows* show the ER and the mitochondria. All values are means ± SD of three independent experiments. Each value is the average of five images per incubation. ∗*p* < 0.05; ∗∗∗*p* < 0.001. GRP75, glucose regulated protein 75; IP3R, inositol 1,4,5 trisphosphate receptor.

Results above demonstrate a role of GRP75 in calcium transport, mitochondrial dysfunction, and apoptosis in MIN6 cells. GRP75 interacts with inositol 1,4,5 trisphosphate receptor (IP3R) on the endoplasmic reticulum and VDAC on the outer mitochondrial membrane at close juxtaposition sites between the ER and mitochondria to catalyze diverse cellular functions. With these, we hypothesized that during apoptotic signaling due to high expression of GRP75, interactions between the ER and the mitochondria might increase; we, therefore, probed ER and mitochondria proximity by labeling these organelles with specific fluorescent proteins (m-cherry for the ER and GFP for mitochondria). ER–mitochondria colocalization significantly increased in the presence of palmitate as indicated by increases in the Pearson coefficient and Mander’s coefficients (Fig. 6*A*), and GRP75 inhibition prevented this event. Overexpression of GRP75 alone led to a significant increase in ER–mitochondria interactions (Fig. 6*B*), all suggesting toward a critical role of GRP75 in ER–mitochondria coupling. As compared with BSA-treated cells, *in-situ* PLA experiments demonstrated a significant increase in IP_3_R1-VDAC1 interactions in palmitate-treated cells as evidenced by increased blobs per nucleus; this increase was prevented in the presence of GRP75 siRNA (Fig. 7*A*) suggesting that GRP75 inhibition prevented palmitate-mediated increase in IP_3_R1-VDAC1 interactions within these cells. Also, GRP75 overexpression alone was sufficient to demonstrate increased IP_3_R1-VDAC1 interaction (Fig. 7*B*). These results were confirmed by transmission electronic microscopy (TEM) analysis, where it was observed that while palmitate (0.4 mM) promoted a significant decrease in the distance between the ER and the mitochondria as compared with BSA-treated cells, this was attenuated in cells transfected with GRP75 siRNA (Fig. 7*C*). Further, as in the *in-situ* PLA, overexpression of GRP75 alone was adequate enough to promote significant decrease in ER–mitochondria proximity (Fig. 7*D*). These data substantiate the role of GRP75 in mediating ER–mitochondria interactions during palmitate-induced cellular dysfunction in MIN6 cells.

### *In vivo* palmitate treatment induces hyperglycemia and impairs glucose and insulin tolerance

**Figure 8:**
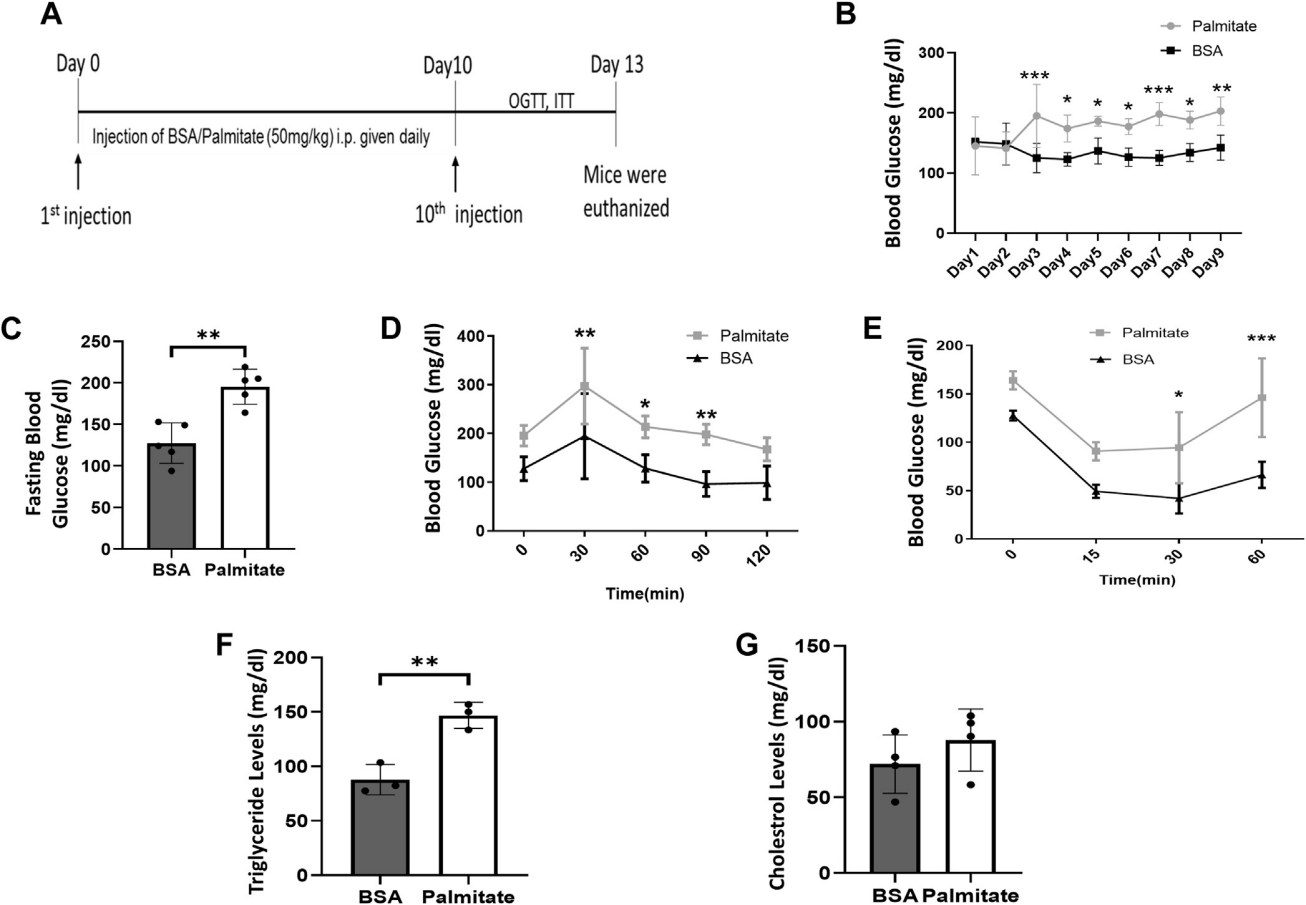
***In vivo* palmitate treatment induces hyperglycemia and hypertriglyceridemia.***A*, wild-type C57BL/6J mice were injected palmitate or BSA, intraperitoneally (i.p.) at a dose of 50 mg/kg. Mice were injected (intraperitoneal) once daily for 10 days and OGTT and ITT were done on Day 11 and 12, respectively, prior to euthanization of mice. *B*, random glucose levels were measured on each day of injection. *C*, fasting glucose levels were measured in BSA or palmitate-injected mice. Mice of both groups were fasted for 12 h, administered glucose at a dose of 2 g/kg body weight by oral gavage, and subjected to OGTT (*D*) and for ITT, were fasted for 6 h, and injected (i.p.) insulin at a dose of 0.75 units/kg body weight (*E*). Serum triglycerides (*F*) and cholesterol (*G*) were measured in animals of both groups. All experiments were performed in at least three animals in each group and values are reported as means ± SD. ∗*p* < 0.05, ∗∗*p* < 0.01, ∗∗∗*p* < 0.001 as compared with BSA-injected animals.

To investigate the effect of palmitate on pancreatic function *in-vivo*, mice were daily injected (i.p.) with 50 mg/kg palmitate for ten consecutive days (Fig. 8*A*). Control mice were injected with BSA. Mice injected with palmitate showed a significant increase in random blood glucose levels from day 3, which persisted for the next 7 days (Fig. 8*B*). Fasting blood glucose levels were also elevated in palmitate-treated mice as compared with BSA-injected mice (Fig. 8*C*). Oral glucose tolerance (OGT) and insulin tolerance (IT) in palmitate-injected mice were significantly impaired as compared with BSA-injected mice (Fig. 8, *D* and *E*). Palmitate elevated serum triglyceride levels, although levels of serum cholesterol did not change as compared with BSA-injected mice (Fig. 8, *F* and *G*). These data suggest that palmitate injection caused hyperglycemia, hypertriglyceridemia, and impaired glucose and insulin tolerance in mice.

### Palmitate treatment induces GRP75 expression and apoptosis in pancreatic islets *in-vivo*

**Figure 9:**
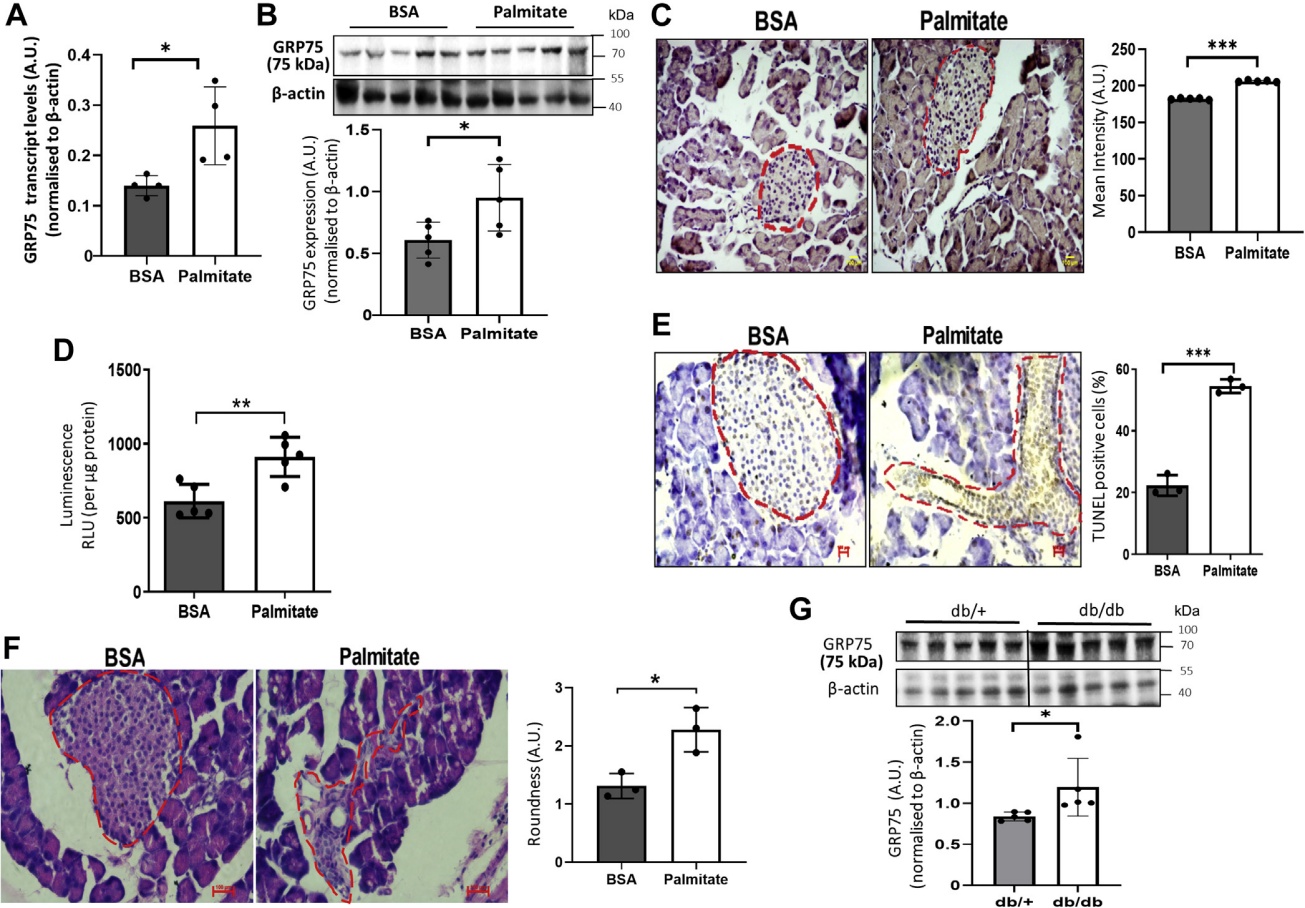
***In vivo* palmitate treatment enhances GRP75 expression.***A*, total RNA was isolated from pancreatic tissues of BSA and palmitate-injected mice and GRP75 transcript levels were assessed by qRT-PCR using specific primers. β-actin was used as normalization control. *B*, pancreatic tissues were homogenized and 30 μg lysate was run on SDS-PAGE and subjected to Western blot analysis using anti-GRP75 antibody. *C*, paraffin-embedded pancreases of BSA and palmitate-injected mice were cut into thin sections (4–5 μm), mounted on poly-L-lysine–coated slides, deparaffinized, and incubated with GRP75 antibody (1:100), followed by incubation with appropriate peroxidase linked secondary antibody (1:250). Detection of GRP75 was done using 3,30–diaminobenzidine (DAB). The sections were counterstained with hematoxylin and visualized in a microscope (20×; scale bars: 100 μm). Quantification of GRP75 within the islets was done using the Image J software. A representative image is shown and the mean intensity of at least three regions within the pancreatic sections of each animal from BSA and palmitate-injected groups is given. Apoptosis within the pancreatic tissues of BSA and palmitate-injected mice were evaluated by caspase 3 activity where lysates (20 μg) were evaluated for caspase activity using Caspase-Glo 3/7 assay kit, and luminescence was measured in a microplate reader (*D*) and by the TUNEL assay where pancreatic tissue sections of BSA and palmitate-injected mice were stained using a TUNEL Assay kit, counterstained in hematoxylin, and visualized in a microscope (40×; scale bars: 100 μm). TUNEL positive cells were counted, normalized to the total number of cells, and the percent of TUNEL positive cells is presented (*E*). RLU, Relative Luciferase Units. *F*, BSA and palmitate-injected mice pancreatic tissues were paraffin-embedded and sections (5 μm) were stained with hematoxylin and eosin and observed under a microscope (40×; scale bars: 100 μm). Morphological quantification of islets was done using the Image-Pro plus tool and the roundness value of the islets is presented. *Red dotted lines* depict the edges of an islet as shown in the pancreatic sections. All experiments were performed in at least three animals in each group. *G*, pancreatic tissues of normal (db/+) and diabetic (db/db) (n = 5) mice were homogenized and 30 μg protein was subjected to Western Blot analyses using GRP75 antibody. β-actin was used as the loading control. Values are means± SD. ∗*p* < 0.05, ∗∗*p* < 0.01, ∗∗∗*p* < 0.001. GRP75, glucose regulated protein 75.

As compared with BSA-injected mice, there was a significant increase in GRP75 levels, both at the transcript and protein levels, in pancreases of palmitate-injected mice (Fig. 9, *A* and *B*). Specifically, islets within the pancreatic tissues exhibited significant but modest increase in GRP75 expression (Fig. 9*C*). As compared with BSA-injected mice, there was increased caspase 3 activity in the pancreatic tissues of mice injected with palmitate (Fig. 9*D*). This was also validated using a TUNEL assay where it was observed that there was a significant increase in TUNEL positive cells in the pancreatic islets of palmitate-treated mice as compared with mice injected with BSA (Fig. 9*E*). Morphologically, islets within the pancreatic tissues of palmitate-injected mice appeared substantially disorganized, disarranged, and deformed as compared with an organized and well-demarcated islet fraction in BSA-injected mice. The roundness of the islets that was obvious and apparent in BSA-injected mice was significantly disturbed in palmitate-treated mice (Fig. 9*F*). When evaluated in a transgenic diabetic (db/db) mice model, it was observed that GRP75 levels are significantly increased in the pancreatic tissues of these mice as compared with normal (db/+) mice (Fig. 9*G*).

All these data suggest that GRP75 plays a potential critical mediatory role in pancreatic islet apoptosis mediated by palmitate, *in vitro* and *in vivo*.

## Discussion

A previous study from our group demonstrated that during pancreatic apoptotic cell death, there is induction of ER stress followed by Ca^2+^ movement from the ER to the mitochondria (29, 30). Interactions between the ER and mitochondria are important for diverse cellular functions such as lipid exchange, calcium homeostasis, and mitochondrial energy generation (5). The ER and mitochondria are coupled by several tethering proteins, and their levels are deregulated in several diseases (31). In this study, we demonstrate that one such ER–mitochondria tethering protein, GRP75, is critical in ER–mitochondria cross talk during palmitate-induced cell death in pancreatic MIN6 cells. GRP75 (HSPA9) is a 75 kDa glucose-regulated protein and belongs to the heat shock 70 kDa protein family of chaperones. It is primarily localized to the mitochondria but is also found in the ER, plasma membrane, and cytoplasmic vesicles (32). Activity and function of GRP75 are determined by its localization in the cell and also by binding to its partners that include p53, FGF-1, IL-1 receptor type1, GRP94, VDAC, NADH dehydrogenase, MPD, Mge1 Tim44, and Tim23 (33, 34, 35, 36, 37, 38). It forms a complex with the ER resident protein IP_3_R and the mitochondrial protein, VDAC to facilitate Ca^2+^ movements (6) and plays roles in cell proliferation, stress response, organellar proteostasis, apoptosis, and vesicular transport of proteins (39).

Our current study shows that GRP75 is significantly upregulated during palmitate-induced cell death in MIN6 insulinoma cells and knockdown of GRP75 was enough to significantly rescue the cells from this effect of palmitate. Palmitate induces apoptosis through multiple processes, including oxidative stress and ER stress. Our study shows that in MIN6 cells, tunicamycin induces ER stress but not GRP75 levels. Such effects of tunicamycin where despite an increase in the levels of ER stress markers, GRP75 levels remain unchanged in muscle cells of mdx mice have been shown by Pauly *et al.* (40). Hence, palmitate-mediated increase in GRP75 levels in the pancreas is possibly not dependent on ER stress induction. While ER and mitochondria dysfunction, independently, is associated with apoptotic cell death, impaired interaction between these organelles is also critical during this event. Most often, this impairment arises due to deregulated tethering between them mediated by altered signatures of MAM proteins. Our data shows that while palmitate significantly increased mitochondrial Ca^2+^ and ROS levels along with impaired mitochondrial membrane potential, GRP75 inhibition clearly prevented these deleterious effects induced by palmitate. Such roles of GRP75 in regulating mitochondrial function, Ca^2+^ and redox homeostasis have been shown during oxidative stress in neuronal cells (41) and during Th1/Th2 imbalance in asthma (42). In adriamycin- or angiotensin II-induced mouse podocyte apoptosis with increased expression of IP_3_R, GRP75, VDAC and MCU, there was enhanced interaction among the IP_3_R-GRP75-VDAC1 complex accompanied by mitochondrial Ca^2+^ overload and increased active caspase-3 levels (43).

While palmitate significantly increased ER–mitochondria tethering in MIN6 cells, GRP75 inhibition significantly prevented this event. In contrast, a previous study showed that palmitate incubation reduced ER–mitochondria interaction in these cells at a dose of 200 μM (18). The reason for this discrepancy might be the lower dose of palmitate used where inspite of affecting ER–mitochondria contact, palmitate did not induce cellular apoptosis. In our study, palmitate increased ER–mitochondria coupling and induced apoptosis in these cells.

GRP75 overexpression alone was sufficient to increase mitochondrial Ca^2+^ levels, impair mitochondrial membrane potential, and induce apoptosis in pancreatic MIN6 cells. Upregulation of GRP75 has been shown to promote ER–mitochondria tethering, increase mitochondrial calcium levels, and enhance ATP generation in injured axons (44). Such increases in cellular GRP75 expression led to increased physical contact between the ER and the mitochondria (17). This was similar to our data on the effects of palmitate in increasing GRP75 levels, increasing ER–mitochondria contact, and impairing mitochondria function; events that were prevented by GRP75 inhibition. GRP75 inhibition decreased ER–mitochondria contact by interfering with the interaction between VDAC1 and IP3R I in Huh7 liver cells (14), consequently impairing insulin stimulated phosphorylation of IR, PKB, and GSK3β. While in skeletal muscle cells, GRP75 inhibition reduced ER–mitochondria coupling resulting in decreased insulin signaling (17), in pancreatic INS-1E cells, GRP75 inhibition disrupted ER–mitochondria interaction and altered GSIS (19). All these suggest that GRP75 is critical during impaired ER–mitochondria–calcium cross talk, consequently leading to apoptotic cell death.

*In vivo* palmitate-injected mice exhibited hyperglycemia, elevated serum triglycerides, and impaired glucose and insulin tolerance. Pancreatic levels of GRP75 were significantly elevated in palmitate-injected mice. There was increased pancreatic islet apoptosis with the islets being disorganized and disarranged in the pancreatic tissues of palmitate-injected mice. Palmitate is a well-accepted contributor of pancreatic apoptosis. The FOXO1 target gene, Pdcd21 and liver X-receptor (LXR) are believed to be a significant mediators of palmitate-induced pancreatic apoptosis (45). Palmitate employs the mitochondrial pathway of cell death through induction of the BH3-only sensitizer, DP5, loss of antiapoptotic Bcl-2 and Mcl-1, and upregulation of the BH3-only activator PUMA, resulting in the activation of Bax/Bak and mitochondrial permeabilization (46). Such lipotoxic stress also blocks the ubiquitin proteasomal system (UPS) and causes apoptosis through induction of ER stress and deregulation of Bcl-2 proteins (47).

Our results identify the MAM protein, GRP75 as a probable major mediator of palmitate-induced impaired ER–mitochondria cross talk and apoptotic cell death in pancreatic islets. Results presented suggest that targeting increased GRP75 levels might be an effective therapeutic strategy to prevent pancreatic islet cell death induced by palmitate, a physiological aberration often associated with diabetes.

## Experimental procedures

### Preparation of palmitate/BSA complex solution

Palmitate/BSA conjugates were prepared as described (48). Briefly, a 20 mM palmitate stock solution was prepared in 0.01 M NaOH by heating at 70 °C. This solution was mixed with 30% fatty acid free BSA at a 3:1 M ratio and used for *in-vivo* injections or diluted in DMEM (high glucose) supplemented with 10% FBS for at a final palmitate concentration of 0.4 mM in *in vitro* experiments.

### Cell culture and incubations

Experiments were done in the mouse islet–derived, insulin-producing cell line, MIN6 and rat insulinoma cells, RINm5F procured from the National Center for Cell Science. They were cultured in DMEM high glucose medium and RPMI media, respectively (Sigma-Aldrich) supplemented with 10% heat-inactivated fetal calf serum (GIBCO Laboratory) at 37 °C and 5% CO_2_. At approximately 60% confluence, cells were treated with either BSA or 0.4 mM palmitate for different time periods (6–36 h). RINm5F cells were treated for with either BSA or palmitate for 24 h. To assess for cell toxicity in MIN6 cells, if any, cell numbers at the end of palmitate incubation (36 h) were evaluated by nuclei staining using DAPI and visualizing in a fluorescent microscope (Leica DM IRB, Leica). Individual nuclei of adherent cells from randomly selected fields of view were counted from at least three separate experiments. Cells were transfected with either the scramble (control) or GRP75 siRNA (25–100 nM, ON Target plus, Dharmacon) using Lipofectamine RNAimax (Invitrogen) according to the manufacturer’s instructions. Cell viability was analyzed using MTT (Sigma-Aldrich), and the absorbance was measured on a plate reader (Tecan) at 570 nm. Values were normalized to the total number of cells. For experiments with palmitate with the scramble or GRP75 siRNA, cells were transfected with either the scramble or GRP75 siRNA and after 24 h, cells were incubated in the presence of palmitate for 24 or 36 h. Wherever mentioned, MIN6 cells were transiently transfected for 48 h with either the control vector or with the GRP75 overexpression vector (2 μg, Origene) using lipofectamine LTX (Invitrogen). The GRP75 insert was subcloned into an inducible pcDNA4/TO/HisA vector (Thermo Fischer) and MIN6 cells grown in six well plates were cotransfected with either the empty vector or the GRP75 inducible vector (2 μg) and the repressor vector, pcDNA6/TR6 (12 μg) using Lipofectamine LTX (Invitrogen). After 24 h, cells were induced with tetracycline (2 μg) and after 48 h, cells were lysed and probed for the levels of GRP75 and cleaved caspase3 by Western Blot analysis and cytosolic and mitochondrial Ca^2+^ levels were measured as described below. For experiments with tunicamycin, MIN6 cells were grown on six-well plates to around 60% confluence and incubated for 8 h with either DMSO or tunicamycin (5 μg/ml) (49).

### RNA isolation and qRT PCR

RNA was isolated from BSA or palmitate-treated cells using TRIzol, reverse transcribed, and PCR amplified for evaluating transcript levels of GRP75, MFN1, MFN2, PACS2, FACL4, and SIGMA1R using gene specific primers (Table 1). Cells were transfected with GRP75 siRNA and the transcript levels of GRP75 mRNA were determined by qRT PCR. Briefly, 1 μg RNA was reverse transcribed using random hexamers (Invitrogen), and the cDNA obtained was amplified using gene specific primers and SYBR Green PCR Master Mix (Applied Biosystems) according to the manufacturer's instructions in a Step One Plus RT PCR system (PE Applied Biosystems). Data was analyzed as described by Pfaffl (50) and is expressed as the fold change in gene expression. All experiments were done in triplicate and β-actin mRNA was taken as normalization control.

**Table 1:** Primer sequences used in qRT-PCR

Sl. No.	Primer	Sequence
1	GRP75 (mouse)	FP: 5′ GCGTCTTATTGGACGACGAT 3′
		RP: 5′ TGGCCCGTAATTTTCTGC 3′
2	FACL4	FP: 5′ TGACGCCCCTCTTTGTAATC 3′
		RP: 5′ GGTGTGTCTGAGGGGACAGT 3′
3	Mitofusin1	FP: 5′ TTTGCCTTGATGCTGATGTC 3′
		RP: 5′ GAAGATGTTGGGCTTGGAGA 3′
4	Mitofusin2	FP: 5′ GCCAGCTTCCTTGAAGACAC 3′
		RP: 5′ GCAGAATTTGTCCCAGAGC 3′
5	Phosphofurin acidic cluster sorting protein 2	FP: 5′ GAACTCCTGTCCGTGGTGAT 3′
		RP: 5′ AGAAGGTCAGAGCCAGGTCA 3′
6	SIGMA1R	FP: 5′ TTTTGAGTCGTGAACCCACA 3′
		RP: 5′ AGAATCAGGGTGATCCATGC 3′
7	d-loop	FP: 5′ CCCAGCTACTACCATCATTCAAGT 3′
		RP: 5′ GATGGTTTGGGAGATTGGTTGATG 3′
8	β2-microglobulin	FP: 5′ GTGACCAAGACTCGTGAGGA 3′
		RP: 5′ ATGCCACAGGTTCATCATGC 3′
9	β-actin	FP: 5′AGCCATGTACGTAGCCATCC 3′
		RP: 5′CTCTCAGCTGTGGTGGTGAA 3′
10	GRP75 (rat)	FP: 5′ CAATGACTCACAGCGACAGG 3′
		RP: 5′ TTTGTCCAGACCGTAAGCCA 3′

FP, forward primer; GRP75, glucose regulated protein 75; RP, reverse primer.

### Western blot analyses

BSA or palmitate-treated cells with or without GRP75 siRNA and cells transfected with the GRP75 overexpression clone were lysed in RIPA buffer (Sigma Chemical Company), and lysates (20–50 μg) were run on SDS-PAGE and evaluated by Western Blot analysis for the protein levels of SIGMA1R (1:2000), PACS2(1:2000), FACL4 (1:2000), IP3R (1:500), MFN1 (1:2000), MFN2 (1:2000), VDAC1 (1:2000), BiP (1:1000), CHOP (1:1000), GRP75 (1:1000), PARP (1:1000), caspase3 (1:500) using specific antibodies (Abcam and Cell Signaling Technology, respectively). DMSO or tunicamycin treated cells were lysed and lysates (30 μg) were similarly probed for the protein levels of BiP, CHOP and GRP75. Immunoreactive bands were detected using the ECL Western Blotting Kit (Pierce, Thermo Scientific). GAPDH, β-actin, vinculin and HSC70 were taken as the loading controls.

### Mitochondrial membrane potential determination

MIN6 cells were cultured in 12-well plates and treated with either BSA or with palmitate (0.4 mM) in the presence of the scramble or GRP75 siRNA. Also, cells were transfected with the empty vector or the GRP75 overexpression vector as mentioned above. Mitochondrial membrane potential was evaluated using TMRE (Invitrogen) according to the manufacturer’s instructions. Briefly, on termination of incubation, cells were treated with 10 nM TMRE in phosphate-buffered saline (PBS) for 30 min at 37 °C and subjected to flow cytometry analysis (FACS LSR; BD Biosciences).

### Intracellular Ca^2+^ concentration measurement

MIN6 cells were cultured in six-well plates and were treated with either BSA or palmitate (0.4 mM) alone or with the scramble or GRP75 siRNA (100 nM). On termination of incubation, cells were washed with PBS and loaded with 2 μM Fluo-3 (cytosolic Ca^2+^) or Rhod-2 (mitochondrial Ca^2+^) and incubated for 15 min at 37 °C. Cells were then washed and fluorescence was measured by flow cytometry (FACS LSR, BD Biosciences). Intracellular calcium levels were also measured by the ratiometric dye, Indo-1AM (Molecular Probes) as described (51). Equal number of cells transfected as above was incubated with 1 μM Indo-1AM (Invitrogen) for 30 min at 37 °C, washed and the fluorescence emission (405 and 485 nm) was measured in a plate reader (TECAN Infinite M200 Pro).The ratio of fluorescence at 405 and 485 nm was used as an index of intracellular calcium concentration. Cytosolic and mitochondrial Ca^2+^ levels were measured in an identical manner in cells transfected with either the vector alone or with the GRP75 overexpression vector.

### Detection of apoptosis by Annexin-PI staining

MIN6 cells were stimulated with palmitate (0.4 mM) for 36 h in the presence and absence of GRP75 siRNA or were transfected with the empty vector or GRP75 overexpression vector, and apoptosis was detected using the FITC Annexin V Apoptosis Detection Kit (Sigma) according to the manufacturer’s instructions. Another set of cells transfected with the scramble or the GRP75 siRNA (100 nM: 48 h) alone was similarly analyzed for the detection of apoptosis. Briefly, cells were harvested by trypsinization and cells was resuspended in the binding buffer containing annexin V-FITC and PI conjugate and incubated for 10 min at room temperature. Cells were then analyzed for apoptosis by flow cytometry (BD FACS Medoly and BD FACS Accuri C6, BD Biosciences). Data was acquired and normalized to equal number of cells.

### ROS (reactive oxygen species) generation

Mitochondrial ROS generation was detected using the MitoSox fluorescent marker (Molecular Probes). MIN6 cells were stimulated with BSA or palmitate (0.4 mM) in the presence and absence of GRP75 siRNA, or cells were transfected with the empty vector or with the GRP75 overexpression vector. On completion of incubation, cells were loaded with 5 μM MitoSox dye (Invitrogen) and incubated for 15 min at 37 °C. Cells were washed and analyzed by flow cytometry (FACS LSR, Becton Dickinson Biosciences).

### Mitochondrial DNA

Total DNA was isolated from MIN6 cells treated with either BSA or palmitate, and GRP75 siRNA and mitochondrial DNA content was quantified using mitochondrial D-loop primers (Table 1) by qRT-PCR as described above. β2-microglobulin (β2M) was used as a normalization control.

### Confocal imaging

For quantification of ER–mitochondria contacts, MIN6 cells were cotransfected with the ER-specific plasmid sec61 mCherry and a mitochondrion specific GFP plasmid (Addgene) together with the empty vector or the GRP75 overexpression vector or with the scramble or GRP75 siRNA and treated with BSA or palmitate (0.4 mM). After incubation, cells were fixed in paraformaldehyde and visualized in a Leica TCS SP8 confocal microscope (Wetzlar) at a magnification of 63× and were analyzed with the Leica Application suite X software (LAS X). Colocalization indices, Pearson’s and Mander’s coefficient were calculated with the colocalization analysis plugin.

### Proximity ligation assay (PLA)

*In situ* PLA was employed to study the proximity between the ER and mitochondria as described by Liu *et al.* (52) using specific probes against the ER protein, IP3R and the mitochondria protein, VDAC1. PLA was performed using the Duolink kit (Duolink -Proximity Ligation kit, Sigma) following the manufacturer's instructions. Briefly, cells grown on chambered glass slides to about 60% confluence were stimulated with palmitate (0.4 mM) for 36 h in the presence and absence of GRP75 siRNA or were transfected with the empty vector or with the GRP75 overexpression vector; after fixation, permeabilization, and blocking, cells were incubated overnight with primary antibodies (VDAC1 (1:200) and IP3R (1:200)), followed by incubation with paired secondary antibodies conjugated with complementary oligonucleotides (anti-rabbit PLUS and anti-mouse MINUS), ligation, amplification, mounting in the DAPI containing Duolink Medium, and imaging in a confocal microscope (Nikon Eclipse Ti2) at a magnification of 60× with a zoom of two times. Negative control PLA experiments were conducted simultaneously. Quantification of PLA signals was done using the Image J software (https://imagej.nih.gov/ij/). All incubations were in triplicate and mean values of at least n = 5 per condition are presented.

### Transmission electron microscopy

MIN6 cells stimulated with palmitate (0.4 mM) in the presence and absence of GRP75 siRNA or transfected with the empty vector or with the GRP75 overexpression vector were visualized for proximity between the ER and the mitochondria using TEM. On termination of incubation, cells were washed and fixed in 2.5% glutaraldehyde for 4 h at room temperature. Subsequent steps of embedding, sectioning, and staining were done at the TEM facility of GB Pant Hospital, New Delhi, India. Single slot grids were visualized in the Talos F200C G2 200 KV Transmission Electron Microscope at a magnification of 13,500× (scale: 500 nm) to visualize the ER and the mitochondria. The distance between the ER and the mitochondria was determined using the Image J software (https://imagej.nih.gov/ij/).

### Pancreatic islet isolation

Pancreatic islets were isolated from adult BALB/c mice housed at the Animal House Facility of the CSIR-Institute of Genomics and Integrative Biology as described (53). Briefly, mice (n = 8) were euthanized and pancreases were aseptically removed, washed with Hanks' balanced salt solution, and digested with collagenase P (0.5 mg/ml) (Sigma–Aldrich). Islets were purified by Ficoll density gradient centrifugation (density: 1.108, 1.096, 1.069 and 1.037) (Sigma-Aldrich) at 800*g* for 15 min at 4 °C, washed and cultured in RPMI 1640 media containing 15% fetal bovine serum, 1% streptomycin–penicillin, and 2 mM L-glutamine at 37 °C under an atmosphere of 5% CO_2_ and 95% O_2_. Equal number of islets were grown in six-well plates and incubated in the presence of BSA or palmitate (0.4 mM) for 24 h. Total RNA was isolated and the levels of GRP75 were assessed by qRT-PCR as described above.

### Animal experiments

Eight to ten-week-old male C57BL6 mice (n = 5) were obtained from the Animal house facility, CSIR-Institute of Genomics and Integrative Biology, New Delhi (India). They were maintained at a 12:12 h light–dark cycle and given *ad libitum* access to food and water. Mice were injected once daily (intraperitoneal) with either BSA or palmitate at a dose of 50 mg/kg body weight for 10 days (54). Daily injections were given at 12 PM and blood was sampled through the tail vein for blood glucose measurements using a One-Touch Glucometer (Lifescan Europe). On completion of the experimental protocol, blood was drawn, serum was immediately separated, animals were euthanized by intraperitoneal injection of thiopentone (10 mg/kg), and pancreatic tissues were collected. For experiments on normal and diabetic mice, 12-week-old male normal (C57BL/KsJ-lepr db/+) and diabetic (C57BL/KsJ lepr db/db) mice were obtained from the Animal House Facility of the CSIR-Central Drug Research Institute, Lucknow, India. The animals were acclimatized for 4 days and the pancreatic tissues were excised and stored at −80 °C until further use. All animal experiments were approved and performed according to the guidelines of the Institutional Animal Ethical Committee (IAEC) of the CSIR-Institute of Genomics and Integrative Biology, New Delhi, India.

### Oral glucose tolerance test (OGTT)

OGTT was performed on overnight fasted mice (12 h). Fasting glucose levels were measured in BSA and palmitate-injected mice. Glucose at a dose of 2 g/kg body weight was administered by oral gavage to both groups of mice, and blood glucose was measured as described above after 30, 60, 90, and 120 min of glucose administration.

### Insulin tolerance test (ITT)

BSA and palmitate-injected mice were fasted for 6 h and given intraperitoneal injections of insulin at a dose of 0.75 units/kg body weight. Blood glucose levels were subsequently measured at 0, 15, 30, and 60 min by bleeding through the tail vein as mentioned above.

### Serum triglyceride and cholesterol determination

Blood samples collected from BSA and palmitate-injected mice were centrifuged at 1300*g* for 10 min at 4 °C, and the serum was analyzed for triglyceride and total cholesterol levels using specific kits (COBAS, Roche) according to the manufacturer’s instructions.

### Quantification of GRP75 levels in pancreatic tissues

Total RNA from pancreatic tissues of BSA or palmitate-injected mice was isolated using TRIzol, reverse transcribed, and transcript levels of GRP75 were quantified by qRT-PCR using specific primers as described above (Table 1). β-actin was used as the normalization control. For Western blot analysis, pancreatic tissues of BSA or palmitate-injected mice or of db/+ or db/db mice were homogenized in RIPA Buffer containing protease inhibitors (Calbiochem), and 30 μg protein from each sample was run on SDS-PAGE and subjected to Western Blot analyses using GRP75 antibody. Immunoreactive bands were detected using the ECL Western Blotting Kit (Pierce, Thermo Scientific). β-actin was used as the loading control.

### Immunohistochemistry

Paraffin-embedded pancreases from BSA or palmitate∖-injected mice were cut into thin sections (4–5 μm), mounted on poly-L-lysine–coated slides, deparaffinized followed by antigen retrieval and protein blocking. Incubation with GRP75 antibody (1:100) was done overnight at 4 °C followed by incubation with appropriate peroxidase linked secondary antibody (1:250) and detection with 3,30–diaminobenzidine (DAB). The sections were counterstained with hematoxylin and visualized at 20× in a microscope (Eclipse, 80i, Nikon). Quantification of GRP75 intensity within the islets was done using the Image J software (https://imagej.nih.gov/ij/).

### Caspase activity assay

Pancreatic tissues from BSA and palmitate-injected mice were lysed in RIPA lysis buffer and lysates (20 μg) were evaluated for caspase activity using Caspase-Glo 3/7 assay kit (Promega) according to the manufacturer’s instructions. Lysates from each sample were incubated with the Caspase Glo reagent for 1 h at room temperature. Luminescence was measured in Infinite 200 pro plate reader (Tecan) and normalized to the protein content.

### Hematoxylin and eosin (H and E) staining

Formaldehyde-preserved pancreatic tissues from BSA and palmitate-treated mice (n = 5) were paraffin-embedded, and 5 μm sections were stained with hematoxylin and eosin. Stained sections were examined by light microscopy, and images were acquired using the NIS-elements imaging software (40×, Eclipse 80*i*, Nikon). Morphological quantification of islets was done using the Image-Pro plus tool (https://www.mediacy.com/imageproplus) where the roundness is calculated by the formula: (perimeterˆ2)/(4.pi. area) within the software, and a value close to 1.0 indicates an absolute circle and larger values indicate noncircular objects.

### TUNEL assay

Apoptosis was assessed in pancreatic tissue sections of BSA and palmitate-injected mice using the DeadEnd Colorimetric TUNEL Assay kit (Promega) according to the manufacturer's protocol. Tissue sections were fixed, deparaffinized, hydrated, and incubated with Proteinase K (1 mg/ml) for 15 min at 37 °C. These were then treated with an equilibration buffer provided within the kit followed by incubation with terminal deoxynucleotidal transferase and biotinylated nucleotides for 1 h at 37 °C. Streptavidin-HRP conjugate was added and incubation was for 30 min at 37 °C ,and then sections were stained with DAB (3,3′-diaminobenzidine) substrate for 5 min. Cells were counter-stained with hematoxylin and visualized in a microscope employing the NIS-elements imaging software (40×, Eclipse 80*i*, Nikon). TUNEL positive cells were counted and normalized to the total number of cells. Results are expressed as percent of TUNEL positive cells.

### Densitometric analyses

Intensities of all Western blot bands were analyzed by Alpha DigiDoc 1201 software (Alpha Innotech Corporation). Same-sized rectangle boxes were drawn surrounding each band, and intensities of the bands were analyzed by the program after subtraction of the background intensity.

### Statistical analyses

Results are means ± SD of at least three independent experiments or at least three animals in each group. Student’s *t* test was employed for differences between two groups and ANOVA followed by a post-hoc test was employed for statistical analysis for comparisons between more than two groups and *p* < 0.05 was taken as statistically significant.

## Data availability

All data generated during the study are contained within the manuscript.

## Conflict of interest

The authors declare that they have no conflicts of interest with the contents of this article.
